# Sulcal morphology in former American football players

**DOI:** 10.1093/braincomms/fcaf345

**Published:** 2025-09-11

**Authors:** Leonard B Jung, Anya S Mirmajlesi, Jared Stearns, Katherine Breedlove, Omar John, Nicholas Kim, Alana Wickham, Yi Su, Hillary Protas, Zachary H Baucom, Fatima Tuz-Zahra, Yorghos Tripodis, Daniel H Daneshvar, Tim L T Wiegand, Tashrif Billah, Ofer Pasternak, Carina Heller, Brian S Im, Shae Datta, Michael J Coleman, Charles H Adler, Charles Bernick, Laura J Balcer, Michael L Alosco, Alexander P Lin, Jeffrey L Cummings, Eric M Reiman, Robert A Stern, Martha E Shenton, Sylvain Bouix, Inga K Koerte, Hector Arciniega, for the DIAGNOSE CTE Research Project, Eric Reiman, Yi Su, Kewei Chen, Hillary Protas, Connie Boker, Michael L Alosco, Rhoda Au, Robert C Cantu, Lindsay Farrer, Robert Helm, Douglas I Katz, Neil Kowall, Jesse Mez, Gustavo Mercier, James Otis, Robert A Stern, Jason Weller, Irene Simkin, Alondra Andino, Shannon Conneely, Courtney Diamond, Tessa Fagle, Olivia Haller, Tennyson Hunt, Nicole Gullotti, Megan Mariani, Brian Mayville, Kathleen McLaughlin, Mary Nanna, Taylor Platt, Surya Pulukuri, Fiona Rice, Madison Sestak, Michael McClean, Yorghos Tripodis, Douglas Annis, Christine Chaisson, Diane B Dixon, Carolyn Finney, Kerrin Gallagher, Kaitlin Hartlage, Jun Lu, Brett Martin, Emmanuel Ojo, Joseph N Palmisano, Brittany Pine, Janani Ramachandran, Sylvain Bouix, Jennifer Fitzsimmons, Alexander P Lin, Inga K Koerte, Ofer Pasternak, Martha E Shenton, Hector Arcinieago, Tashrif Billah, Elena Bonke, Katherine Breedlove, Eduardo Coello, Michael J Coleman, Leonhard Jung, Huijun Liao, Maria Loy, Elizabeth Rizzoni, Vivian Schultz, Annelise Silva, Brynn Vessey, Tim L T Wiegand, Sarah Banks, Charles Bernick, Jason Miller, Aaron Ritter, Marwan Sabbagh, Raelynn de la Cruz, Jan Durant, Morgan Golceker, Nicolette Harmon, Kaeson Kaylegian, Rachelle Long, Christin Nance, Priscilla Sandoval, Robert W Turner, Kenneth L Marek, Andrew Serrano, Charles H Adler, David W Dodick, Yonas Geda, Jennifer V Wethe, Bryce Falk, Amy Duffy, Marci Howard, Michelle Montague, Thomas Osgood, Debra Babcock, Patrick Bellgowan, Laura Balcer, William Barr, Judith Goldberg, Thomas Wisniewski, Ivan Kirov, Yvonne Lui, Charles Marmar, Lisena Hasanaj, Liliana Serrano, Alhassan Al-Kharafi, Allan George, Sammie Martin, Edward Riley, William Runge, Jeffrey L Cummings, Elaine R Peskind, Elizabeth Colasurdo, Daniel S Marcus, Jenny Gurney, Richard Greenwald, Keith A Johnson

**Affiliations:** Department of Rehabilitation Medicine, NYU Grossman School of Medicine, New York, NY 10016, USA; Psychiatry Neuroimaging Laboratory, Brigham and Women’s Hospital, Harvard Medical School, Boston, MA 02145, USA; cBRAIN, Department of Child and Adolescent Psychiatry, Psychosomatics, and Psychotherapy, University Hospital, Ludwig-Maximilians-Universität, Munich, Bavaria 80336, Germany; Department of Neurosurgery, University Hospital, Ludwig-Maximilians-Universität, Munich, Bavaria 80336, Germany; Department of Rehabilitation Medicine, NYU Grossman School of Medicine, New York, NY 10016, USA; NYU Langone Concussion Center, NYU Langone Health, New York, NY 10016, USA; Department of Rehabilitation Medicine, NYU Grossman School of Medicine, New York, NY 10016, USA; NYU Langone Concussion Center, NYU Langone Health, New York, NY 10016, USA; Center for Clinical Spectroscopy, Department of Radiology, Brigham and Women’s Hospital, Harvard Medical School, Boston, MA 02115, USA; Department of Rehabilitation Medicine, NYU Grossman School of Medicine, New York, NY 10016, USA; Psychiatry Neuroimaging Laboratory, Brigham and Women’s Hospital, Harvard Medical School, Boston, MA 02145, USA; Psychiatry Neuroimaging Laboratory, Brigham and Women’s Hospital, Harvard Medical School, Boston, MA 02145, USA; Banner Alzheimer's Institute, Arizona State University, and Arizona Alzheimer's Consortium, Phoenix, AZ 85006, USA; Banner Alzheimer's Institute, Arizona State University, and Arizona Alzheimer's Consortium, Phoenix, AZ 85006, USA; Department of Biostatistics, Boston University School of Public Health, Boston, MA 02118, USA; Department of Biostatistics, Boston University School of Public Health, Boston, MA 02118, USA; Department of Biostatistics, Boston University School of Public Health, Boston, MA 02118, USA; Department of Physical Medicine and Rehabilitation, Harvard Medical School, Boston, MA 02115, USA; Department of Physical Medicine and Rehabilitation, Massachusetts General Hospital, Boston, MA 02114, USA; Department of Physical Medicine and Rehabilitation, Spaulding Rehabilitation Hospital, Boston, MA 02129, USA; Psychiatry Neuroimaging Laboratory, Brigham and Women’s Hospital, Harvard Medical School, Boston, MA 02145, USA; cBRAIN, Department of Child and Adolescent Psychiatry, Psychosomatics, and Psychotherapy, University Hospital, Ludwig-Maximilians-Universität, Munich, Bavaria 80336, Germany; Psychiatry Neuroimaging Laboratory, Brigham and Women’s Hospital, Harvard Medical School, Boston, MA 02145, USA; Psychiatry Neuroimaging Laboratory, Brigham and Women’s Hospital, Harvard Medical School, Boston, MA 02145, USA; Department of Radiology, Brigham and Women’s Hospital, Harvard Medical School, Boston, MA 02115, USA; Department of Psychiatry, Massachusetts General Hospital, Boston, MA 02114, USA; Masonic Institute for the Developing Brain, University of Minnesota, Minneapolis, MN, USA; Department of Pediatrics, University of Minnesota, Minneapolis, MN, USA; Department of Psychological and Brain Sciences, University of California, Santa Barbara, CA, USA; Department of Rehabilitation Medicine, NYU Grossman School of Medicine, New York, NY 10016, USA; NYU Langone Concussion Center, NYU Langone Health, New York, NY 10016, USA; NYU Langone Concussion Center, NYU Langone Health, New York, NY 10016, USA; Department of Neurology, NYU Grossman School of Medicine, New York, NY 10016, USA; Psychiatry Neuroimaging Laboratory, Brigham and Women’s Hospital, Harvard Medical School, Boston, MA 02145, USA; Department of Neurology, Mayo Clinic College of Medicine, Mayo Clinic Arizona, Scottsdale, AZ 85259, USA; Cleveland Clinic Lou Ruvo Center for Brain Health, Las Vegas, NV 89106, USA; Department of Neurology, University of Washington, Seattle, WA 98195, USA; Department of Neurology, NYU Grossman School of Medicine, New York, NY 10016, USA; Department of Population Health, NYU Grossman School of Medicine, New York, NY 10017, USA; Department of Ophthalmology, NYU Grossman School of Medicine, New York, NY 10017, USA; Department of Neurology, Boston University Alzheimer’s Disease Research Center and CTE Center, Boston University Chobanian & Avedisian School of Medicine, Boston, MA 02118, USA; Center for Clinical Spectroscopy, Department of Radiology, Brigham and Women’s Hospital, Harvard Medical School, Boston, MA 02115, USA; Department of Radiology, Brigham and Women’s Hospital, Harvard Medical School, Boston, MA 02115, USA; Chambers-Grundy Center for Transformative Neuroscience, Pam Quirk Brain Health and Biomarker Laboratory, Department of Brain Health, School of Integrated Health Sciences, University of Nevada, Las Vegas, Las Vegas, NV 89154, USA; Banner Alzheimer's Institute, Arizona State University, and Arizona Alzheimer's Consortium, Phoenix, AZ 85006, USA; Department of Psychiatry, University of Arizona, Phoenix, AZ 85004, USA; Department of Psychiatry, Arizona State University, Phoenix, AZ 85008, USA; Neurogenomics Division, Translational Genomics Research Institute and Alzheimer’s Consortium, Phoenix, AZ 85004, USA; Department of Neurology, Boston University Alzheimer’s Disease Research Center and CTE Center, Boston University Chobanian & Avedisian School of Medicine, Boston, MA 02118, USA; Department of Anatomy and Neurobiology, Boston University Chobanian & Avedisian School of Medicine, Boston, MA 02118, USA; Department of Neurosurgery, Boston University Chobanian & Avedisian School of Medicine, Boston, MA 02118, USA; Psychiatry Neuroimaging Laboratory, Brigham and Women’s Hospital, Harvard Medical School, Boston, MA 02145, USA; Department of Radiology, Brigham and Women’s Hospital, Harvard Medical School, Boston, MA 02115, USA; Department of Psychiatry, Massachusetts General Hospital, Boston, MA 02114, USA; Department of Software Engineering and Information Technology, École de Technologie Supérieure, Université du Québec, Montréal, QC H3C 1K3, Canada; Psychiatry Neuroimaging Laboratory, Brigham and Women’s Hospital, Harvard Medical School, Boston, MA 02145, USA; cBRAIN, Department of Child and Adolescent Psychiatry, Psychosomatics, and Psychotherapy, University Hospital, Ludwig-Maximilians-Universität, Munich, Bavaria 80336, Germany; Department of Radiology, Brigham and Women’s Hospital, Harvard Medical School, Boston, MA 02115, USA; Graduate School of Systemic Neurosciences, Ludwig-Maximilians-Universität, Munich, Bavaria 82152, Germany; German Center for Child and Adolescent Health (DZKJ), Partner Site Munich, Munich, Bavaria 82152, Germany; Department of Rehabilitation Medicine, NYU Grossman School of Medicine, New York, NY 10016, USA; NYU Langone Concussion Center, NYU Langone Health, New York, NY 10016, USA

**Keywords:** neuroimaging, structural MRI, sports-related head injury, repetitive head impact, former American football players

## Abstract

Repetitive head impacts are associated with structural brain changes and an increased risk for chronic traumatic encephalopathy, a progressive neurodegenerative disease that can only be diagnosed after death. Chronic traumatic encephalopathy is defined by the abnormal accumulation of phosphorylated tau protein, particularly at the depths of the superior frontal sulci, suggesting that sulcal morphology may serve as a relevant structural biomarker. Contact sport athletes, such as former football players, are at elevated risk due to their prolonged exposure to repetitive head impacts. Cortical atrophy linked to underlying tau accumulation may result in shallower and wider sulci, potentially making sulcal morphology an imaging marker for identifying individuals at risk for this disease. This study investigated sulcal morphological differences in former football players and examined associations with age, football-related exposure, clinical diagnosis of traumatic encephalopathy syndrome, levels of certainty for chronic traumatic encephalopathy pathology, neuropsychological performance, and positron emission tomography imaging using flortaucipir. We analysed structural magnetic resonance imaging data from 169 male former football players (mean age 57.2 (8.2) years, range 45–74) and 54 age-matched, unexposed asymptomatic male controls (mean age 59.4 (8.5) years, range 45–74). Sulcal depth and width were quantified using the CalcSulc, focusing on two regions in each hemisphere commonly affected by chronic traumatic encephalopathy pathology: the superior frontal and occipitotemporal sulci. Generalized least squares models were used to assess group differences and interactions with age and football exposure variables, including age of first exposure, total years played, and cumulative head impact exposure. An analysis of covariance evaluated relationships between sulcal morphology, clinical measures, and flortaucipir uptake, adjusting for age, race, body mass index, education, imaging site, apolipoprotein E4 status, and total intracranial volume. Former football players demonstrated significantly shallower sulcal depth in the left superior frontal sulcus compared to unexposed controls. Earlier age of first exposure and longer football careers were associated with greater widening of the left occipitotemporal sulcus. Higher cumulative head impact exposure was linked to reduced sulcal depth in the left superior frontal region. However, sulcal morphology was not associated with clinical diagnosis, levels of certainty, neuropsychological test performance, or flortaucipir imaging. These findings suggest that sulcal morphology may reflect cumulative exposure to repetitive head impacts, particularly in brain regions vulnerable to chronic traumatic encephalopathy pathology. Future ante- and post-mortem validation studies are needed to determine whether sulcal morphology can serve as a reliable *in vivo* biomarker of risk.

## Teaser

This study demonstrates that former football players show altered sulcal morphology in brain regions vulnerable to repetitive head impacts. Shallower and wider sulci were associated with earlier age of first exposure and longer play duration. Findings suggest sulcal morphology may serve as a structural marker of cumulative head impact exposure.

## Graphical Abstract

**Graphical Abstract:**
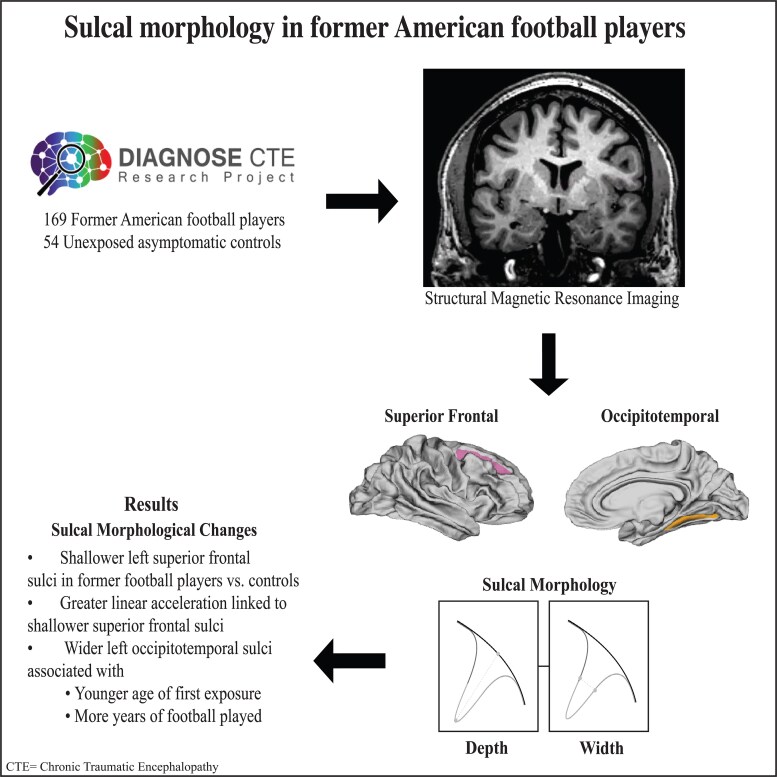

## Introduction

Repetitive head impacts (RHI) are associated with cognitive, functional and behavioural impairments and increase the risk of developing neurodegenerative diseases, including chronic traumatic encephalopathy (CTE).^1-3^ CTE has been linked to participation in contact and collision sports, such as American football, where RHI is frequent.^4-9^ Understanding the long-term consequences of RHI and identifying clinical and biomarker features indicative of CTE *in vivo* is critical for advancing early detection, refining diagnostic criteria and developing targeted interventions to mitigate disease progression.

Currently, one approach aimed at identifying CTE in living individuals relies on the clinical classification of symptoms outlined in the 2021 National Institute of Neurological Disorders and Stroke (NINDS) consensus diagnostic criteria for Traumatic Encephalopathy Syndrome (TES).^10^ Following a TES diagnosis, a level of certainty for CTE pathology (suggestive, possible, probable or definite CTE with TES) can be assigned. However, this classification relies solely on the progression of clinical symptoms and supportive clinical features without the incorporation of objective biomarkers.^10^ Identifying and validating supportive biomarkers, such as neuroimaging and blood-based markers, may improve the accuracy of CTE diagnosis and further elucidate the pathological consequences of RHI. Among potential neuroimaging biomarkers, positron emission tomography (PET) has emerged as a promising tool for detecting pathological changes associated with CTE. Preliminary evidence suggests that flortaucipir PET binding is associated with CTE pathology, but its specificity for tau aggregation in CTE remains under investigation.^11,12^ Further research is required to determine the relationship between flortaucipir retention and structural brain changes in regions known to be affected by CTE pathology.

One possible approach for identifying *in vivo* biomarkers of RHI and CTE involves examining neuroanatomical alterations in individuals at increased risk, such as former American football players.^1-3,13-15^ Structural magnetic resonance imaging (MRI) provides an opportunity to investigate cortical features that may serve as surrogate markers of neurodegeneration in CTE. Notably, postmortem studies have demonstrated that phosphorylated tau (p-tau) aggregates in CTE are predominantly localized to the depths of the frontal and temporal sulci.^2,3,16,17^ As the disease progresses, the accumulation of tau pathology and neurodegeneration leads to cortical atrophy, potentially resulting in sulcal widening and shallowing where such structural alterations may serve as quantifiable markers of disease progression.^4,6,18^ Unlike traditional morphometric metrics such as cortical thickness, volume, or surface area, sulcal measures may provide enhanced sensitivity to these focal pathological changes, particularly given the preferential accumulation of tau in sulcal depths. Sulcal widening and shallowing have also been observed in other neurodegenerative conditions, including Alzheimer's disease, where these morphological changes are frequently detected in medial temporal regions.^19-22^ Given the distinct localization of tau pathology in CTE, it is plausible that sulcal morphological alterations, specifically, shallower and wider sulci in the frontal and temporal cortices, may serve as indicators of CTE risk in individuals with a history of RHI.^2,3,18,23,24^ These structural changes may reflect underlying neurodegenerative processes, including tau-mediated neurotoxicity, neuroinflammation and cortical atrophy, which are hypothesized to contribute to disease onset and progression.^2-5,18,23,25,26^ Investigating sulcal morphology in at-risk populations may provide critical insights into the detection and staging of CTE, ultimately informing biomarker development and potential intervention strategies.

In this study, we investigated four main hypotheses. First, we assess *in vivo* sulcal morphometric changes in former American football players, focusing on the superior frontal and temporal lobes—regions implicated in postmortem CTE pathology.^3,4,16,26,27^ We hypothesize that these individuals will exhibit shallower sulcal depth and wider sulci compared to unexposed asymptomatic controls. Second, we investigate the relationship between sulcal morphology, age and RHI-related exposure factors [age of first exposure, total years in football, cumulative head impact index (CHII) measures, including frequency, linear acceleration and rotational force]. We hypothesize that greater age and RHI exposure will be associated with increased sulcal widening and shallowing, suggesting a dose–response relationship contributing to the development of CTE pathology. Third, we hypothesize that former American football players with TES will exhibit distinct sulcal morphological differences compared to those without TES. Additionally, we expect that football players with higher levels of certainty for CTE pathology will show increased sulcal widening and shallowing, further linking clinical symptoms and diagnosis to underlying neuroanatomical changes associated with CTE. Finally, we hypothesize that these sulcal alterations will correlate with higher flortaucipir uptake on PET imaging, suggesting a potential relationship between structural changes and tau pathology in CTE.

## Materials and methods

### Study design and participants

This study is part of the Diagnostics, Imaging, And Genetics Network for the Objective Study and Evaluation of Chronic Traumatic Encephalopathy (DIAGNOSE CTE) Research Project.^28^ Data were collected from September 2016 to February 2020 at Boston University Chobanian & Avedisian School of Medicine (MRI data collection at Brigham and Women’s Hospital), New York University Langone Medical Center, Cleveland Clinic Lou Ruvo Center for Brain Health in Las Vegas and the Mayo Clinic in Arizona (PET data collection at Banner Alzheimer’s Institute). All study sites received approval from their Institutional Review Board, and all participants provided written informed consent before enrolment. The DIAGNOSE CTE Research Project employed an open recruitment strategy to ensure an unbiased approach to recruitment.^28^ The college football group includes individuals who played ≥6 years of organized football, including ≥3 years at the collegiate varsity level, and did not participate in contact or collision sports after college. The professional football group includes individuals with ≥12 years of organized play, including ≥3 years in both college and the National Football League (NFL). Only those who played high-exposure positions (offensive/defensive linemen, linebackers, running backs, receivers or defensive backs) are eligible; quarterbacks and kickers are excluded due to their lower exposure risk. All football-exposed participants must be English-speaking males aged 45–74 and meet general health and safety criteria for imaging and biomarker procedures. The unexposed asymptomatic control group comprises individuals with no history of participation in organized contact/collision sports and military combat. They must be asymptomatic at screening (no current mood, behavioural, cognitive or functional complaints), have no history of traumatic brain injury (TBI), psychiatric illness or cognitive impairment, and possess at least 2 years of post-secondary education. We provide detailed study inclusion criteria in Supplementary Fig 1 and Supplementary Table 1. This transparent approach helped mitigate potential biases in participant selection and ensured a more representative sample for the study.

A total of 240 participants were enrolled in the study, including 180 former American football players (120 professional football players, 60 former college players) and 60 unexposed asymptomatic control participants with no history of RHI, TBI or prior participation in organized sports. Data from 11 former American football players and two unexposed asymptomatic controls were excluded from the analyses because of poor quality and/or incomplete structural MRIs. Furthermore, at follow-up, four controls reported prior RHI exposure/head injury or long-standing psychiatric illness and were therefore excluded from the analyses. The final sample consisted of 169 former American football players (114 former professional and 55 former college players) and 54 unexposed asymptomatic controls who had no history of RHI exposure, TBI, or long-standing psychiatric illness.

### Sample characteristics

Semi-structured interviews and online questionnaires were used to acquire information about demographics (e.g. age, education, race and ethnicity), clinical history, athletic history, military involvement and TBI history [number, type, severity and timing of injuries, as well as exposure to RHI across different contexts (e.g. sports, military, occupational)]. Age was analysed as a continuous variable. Education was defined by total years in school (e.g. high school graduate = 12 years). Race and ethnicity were coded using the Office of Management and Budget Directive 15 definitions of racial and ethnic categories, and participants could select more than one race or ethnicity. Body mass index (BMI) was calculated using the participant's height and weight. Apolipoprotein E *ε*4 (APOE4) genotyping was performed for each participant. See Table 1 for all cohort characteristics and Supplementary Fig 1 and Supplementary Table 1 for recruitment information.

**Table 1:** Cohort demographics

	Former football players (*n* = 169)	Unexposed asymptomatic controls (*n* = 54)
Primary demographics		
Age	57.2 y (8.2), [45–74 y]	59.4 y (8.5), [45–74 y]
BMI kg/m^2^	32.7 (4.7), [22.8–47.4]	31 (4.6), [23.7–43.5]
Education	16.7 y (1.5), [15–27 y]	17.2 y (3.4), [13–30 y]
Apolipoprotein 4 carriers^a^	47 (28%)	10 (18.5%)
Race		
White	108 (64%)	34 (63%)
Black/African American	58 (34%)	19 (35.2%)
American Indian/Alaska Native	0 (0%)	0 (0%)
Asian	0 (0%)	0 (0%)
Native Hawaiian/Other Pacific Islander	0 (0%)	1 (1.8%)
Multiple Races	3 (2%)	0 (0%)
Exposure to RHIs		
Total years in Football	16y (4.3), [6–25 y]	
Age of first exposure	11.1 y (2.8), [4–18 y]	
Cumulative head impact index seasons		
Frequency	10 895 (4691), [3560–28 020]	
Linear acceleration (g)	228 327 (73 362), [79 212–446 257]	
Rotational force (rad/s^2^)	18 302 568 (6 448 603), [6 053 874–44 072 194]	
PET SUVR		
Superior frontal	1.1(0.09)	
Superior temporal	1.02 (0.06)	
Traumatic encephalopathy syndrome		
Traumatic encephalopathy Syndrome diagnosis (%)^b^	107 (63%)	
Neuropsychological evaluations		
MoCA	24.7 (3.4) [11–30]	
NAB list learning^c^	5.1 (3.0) [0–11]	
Trails A	31.3 (13.3) [12.2–118]	
Trails B	82.1 (47.2) [29–300]	

Overview of cohort characteristics including demographics of 169 former football players and 54 unexposed asymptomatic control participants. Values represent mean, (standard deviation) [range].

RHI, repetitive head impacts; y, years.

^a^Apolipoprotein 4 carrier analysis was only available for 210 participants.

^b^Traumatic Encephalopathy Syndrome diagnosis is missing for one participant.

^c^One participant is missing the NAB List Learning Long Delay, and one other participant was missing all neuropsychological evaluations.

### MRI acquisition

All study participants underwent a head MRI scan at one of the four sites or their corresponding imaging centres. All scans followed the same imaging acquisition protocol, with total acquisition time lasting approximately 50–60 min, as part of a multi-sequence neuroimaging protocol and used the same 3T scanner model (Siemens Magnetom Skyra, Erlangen, Germany; software version VE11) with a 20-channel head coil across the four sites. The protocol included structural, diffusion and functional MRI scans. Relevant to this study is the high resolution (1 × 1 × 1 mm^3^) 3D T1-weighted (T1w) magnetization-prepared-rapid-gradient-echo (MPRAGE) sequence (inversion time = 1100 ms, TR = 2530 ms, TE = 3.36 ms, 7-degree flip angle, 256 FOV) and the high resolution (1 × 1 × 1 mm^3^) and the 3D T2-weighted (T2w) Sampling-Perfection-with-Application-optimized-Contrasts-by-using-flip-angle-Evolution (SPACE) sequence (TR = 3200 ms, TE = 412 ms, 256 FOV).

### MRI processing and calculation of sulcal morphology

**Figure 1:**
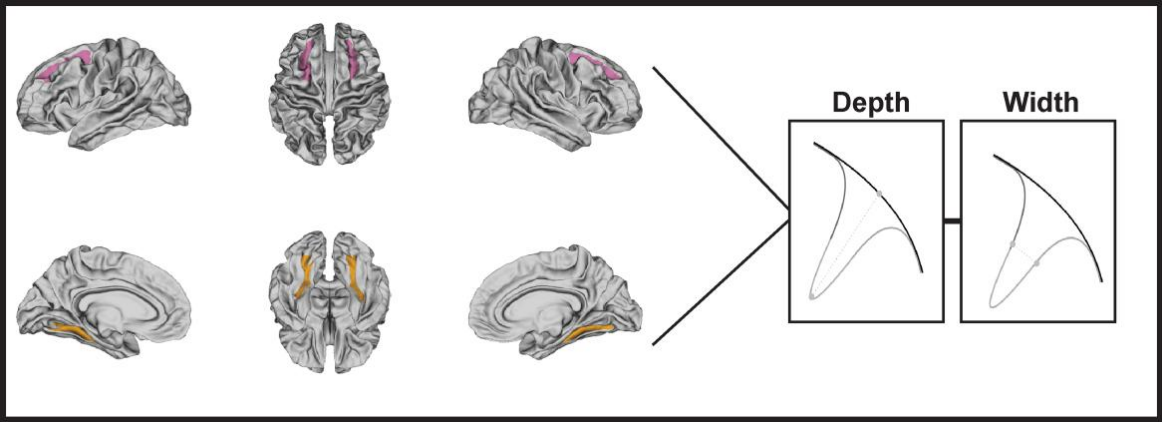
**Illustration of the sulcal morphological regions with depictions of width and depth.** The superior frontal area is highlighted in the top panel, while the occipitotemporal areas are shaded in the bottom panel. The image on the right provides a concise overview of how CalcSulc calculates depth and width.

The raw images were visually inspected for completeness, distortion and motion artefacts using 3D Slicer (version 4.10, Surgical Planning Laboratory, Brigham and Women’s Hospital, Boston, MA, USA). Brain masking was performed for all T1w and T2w scans using custom tools developed by the Psychiatry Neuroimaging Laboratory^29,30^ and further processed with FreeSurfer v7.1. to generate volumetric segmentations and parcellations of cortical and subcortical structures according to the Destrieux atlas.^31-37^ All FreeSurfer segmentations were quality controlled by blinded raters. Following this, we calculated the local gyrification index using FreeSurfer.^38^ Calculations of sulcal width and depth were then extracted using the CalcSulc toolbox in MATLAB (The MathWorks, Natick, MA).^39^ Sulcal morphology measures were extracted from the CalcSulc output, encompassing eight regions of interest in both the left and right hemispheres, resulting in 16 regions. However, six of the eight regions are not usually affected by CTE neuropathology. Thus, to investigate the regions implicated in CTE pathology, our analysis focused a priori on two anatomical regions, the superior frontal and the medial occipitotemporal and lingual sulcus (closest approximation to the temporal lobe) regions in both the left and right hemispheres resulting in a total of four regions of interest; see Fig. 1 for region locations; image was created using *fsbrain.*^40^ In Supplementary Tables 2 and 3, we provide estimates and confidence intervals for the depth and width of sulci across a total of 16 regions as calculated using CalcSulc.

### PET acquisition

Participants underwent a PET scan utilizing a flortaucipir tracer (18F-AV-1451). The tracer dose was obtained from Avid Radiopharmaceuticals (Philadelphia, PA, USA). Readers are directed to the methods paper from the DIAGNOSE CTE Research Project^28^ for a comprehensive overview of PET imaging details. In this study, the application of flortaucipir was conducted under an Investigational New Drug (IND #131391) obtained from the U.S. Food and Drug Administration. The flortaucipir protocol involved a 370 MBq (10 mCi) bolus injection, and 80 min later, the participant underwent a continuous dynamic 20-min brain scan, consisting of four frames with a duration of 5 min each.

### PET processing

Flortaucipir PET images were processed using the PET Unified Pipeline, involving scanner harmonization, motion correction, frame summation, PET to MRI co-registration and standard uptake value ratio (SUVR) estimation with the cerebellum cortex as the reference region.^41,42^ Regional SUVR values were provided for each FreeSurfer-defined volume using the Desikan–Killiany Atlas. The process included partial volume correction using the regional spread function. Spatial normalization was achieved using Statistical Parametric Mapping 12 (SPM12) with T1w images, transforming flortaucipir PET images to the MNI template space.^41^ Due to variations between the atlas outputs from CalcSulc and PET images, we opted for the closest approximations from the PET Desikan–Killiany atlas to the CalcSulc Destrieux Atlas. Accordingly, we chose the SUVR of the superior frontal and superior temporal regions of the Desikan–Killiany Atlas for analysis. All analyses evaluating PET data only included the sample of former American football players. Exclusion criteria for PET analyses were missing data. Consequently, 10 participants from our sample lacked PET imaging, and these 10 participants were excluded from any analysis involving flortaucipir PET.

### Exposure to RHI

Exposure to American football was evaluated based on self-reported age at first exposure to tackle football as well as the total years of active football play. Additionally, we calculated CHII scores for the former American football player group. The rationale behind utilizing CHII is to account for the variability in head impact patterns over a career in American football. CHII scores are calculated using the total seasons of active football play, the playing positions at each career level, and the estimated head impact profile of the playing position at each level, based on previous studies of helmet accelerometry.^9^ The scores are used to estimate the frequency (CHII), linear acceleration (CHII-G) and rotational force (CHII-R) endured over a career in American football.^9^ Higher CHII scores reflect greater exposure to RHI.

### Evaluation of TES and provisional levels of certainty for CTE pathology

All participants were evaluated for a diagnosis of TES based on a multidisciplinary diagnostic consensus conference using the 2021 NINDS Consensus Diagnostic Criteria.^10^ The DIAGNOSE CTE consensus panel evaluated the participant’s medical history; RHI exposure; self- and informant-reported complaints of cognitive, mood and/or behaviour problems, as well as functional dependence status; neurological/motor evaluation findings; and standardized neuropsychological and neuropsychiatric test results. A diagnosis of TES (yes/no) was then adjudicated based on: (i) substantial exposure to RHI; (ii) core clinical features involving *cognitive impairment* (yes/no) and/or *neurobehavioural dysregulation* (yes/no), (iii) evidence of *progressive worsening* of clinical symptoms (yes/no) and (iv) absence of another disease or condition that could account for core clinical features. See Table 1 for details.

Provisional levels of certainty for CTE pathology (suggestive, possible, probable, definite CTE with TES) were then determined through a stepwise evaluation alongside a TES diagnosis. This evaluation considers RHI exposure, distinct clinical characteristics and a collection of supportive features.^10^ Of note, none of our football players had a provisional level of certainty for CTE pathology that was classified as definite CTE with TES, as this would require postmortem evaluation. See Table 1 for a breakdown of levels of certainty for CTE pathology among our football players.

### Objective neuropsychological evaluation

As a post-hoc analysis, we examined objective neuropsychological test performance exclusively in our former American football players. Each participant completed an in-person baseline neuropsychological assessment, which included a standardized battery of paper-and-pencil tests administered by a trained examiner.^28^ A comprehensive list of the cognitive domains assessed, specific tests administered, and individual performance metrics can be found in Alosco *et al*.^43^ Our analysis cantered on the three most affected cognitive domains—learning and memory, attention and psychomotor speed and executive function. To evaluate performance, we extracted raw scores from four key measures: the Montreal Cognitive Assessment (MoCA), the Neuropsychological Assessment Battery (NAB) List Learning Long Delay,^44^ Trail Making Test Part A^45^ and Trail Making Test Part B.^45^ A summary of the raw data is provided in Table 1.

### Statistical analysis

#### Group level differences and interactions

To examine group-level differences in demographic variables between former American football players and unexposed asymptomatic control participants, we conducted an independent Welch’s *t*-test for continuous variables (age, BMI and education) and applied a chi-square test for categorical variables (race and APOE4 gene status).

We used a generalized least squares model with sulcal width and depth as respective outcome variables to compare group-level differences using customized code in R Studio. In this model and the ANCOVA, we controlled for age,^46-48^ BMI,^49-51^ race,^48,52^ education in years,^53-56^ imaging site, total intracranial volume^57,58^ and APOE4 allele status (presence or absence).^59,60^ We carefully selected these covariates as they have either been shown to affect neurodegenerative processes and/or MRI-based imaging analyses. Throughout our analyses, we report 95% confidence intervals (CIs), and all *P*-values were corrected for multiple comparisons across the full set of regions of interest and outcome variables. Specifically, for each family of tests (i.e. all regions of interest analysed for a given outcome), we controlled the false discovery rate (FDR) using the Benjamini & Hochberg method. Only FDR-adjusted *P*-values less than 0.05 were considered statistically significant. This approach ensures that the correction was applied across all ROIs within each outcome, not just within individual regions. Using the generalized least square model, we also tested interactions with age and exposure factors (age of first exposure, total years of football played, CHII frequency, linear acceleration and rotational forces) and sulcal width and depth as respective outcome variables. We corrected for total years in football as an additional covariate when testing interactions between the age of first exposure and sulcal morphology.

#### Group-level differences in TES

We compared sulcal morphology in former American football players with and without a diagnosis of TES using the generalized least squares model, correcting for the same covariates listed above. Furthermore, we compared sulcal morphology in former American football players across the different levels of certainty for CTE pathology using an ANCOVA.

#### Associations with neuropsychological evaluations

To investigate the relationship between our regions of interest linked to CTE pathology in postmortem studies and individual neuropsychological performance, we conducted a linear regression analysis. This analysis examined scores from the MoCA, NAB List Learning Long Delay, Trail Making Test Part A and Trail Making Test Part B while accounting for key covariates, including age, race, BMI, education, imaging site, APOE4 gene status and total intracranial volume. To control for multiple comparisons, we applied the Benjamini & Hochberg correction. The analysis was restricted to former American football players, with two participants excluded due to missing data.

#### Flortaucipir-PET

For assessing the relationship between sulcal width and depth with flortaucipir SUVR values, we conducted a linear regression while controlling for the aforementioned covariates: age, BMI, race, education in years, imaging site, total intracranial volume and APOE4 allele status. The resulting *P*-values were adjusted for multiple comparisons using the Benjamini & Hochberg method. For the analysis of exposure factors, TES, and flortaucipir, we evaluated only the former American football player group.

## Results

### Demographics

Using a Welch two-sample *t*-test, we detected differences in BMI between our former American football players and unexposed asymptomatic controls (*t*(102) = 2.6, mean difference = 1.8, 95% CI [0.5, 3.2], *P* = 0.01), signifying a higher BMI in the football player group. No additional group differences were evident.

### Group differences in sulcal morphology

Using the generalized least squares model, we tested for differences in sulcal width and depth of the superior frontal and occipitotemporal areas between former American football players and the unexposed asymptomatic control group. This model identified shallower sulcal depth in former American football players in the left hemisphere superior frontal region (95% CI [−0.96, −0.07], *P* = 0.04). No other main effects reached significance; all *P*’s > 0.2; see Table 2, and Supplementary Tables 2 and 3 for a summary of all findings.

**Table 2:** Group-level differences for sulcal morphology

		Left				Right			
Region of interest		Estimate	SD	95% CI	*P* Value	Estimate	SD	95% CI	*P* Value
**Group-Level (Football × Control)**									
**Depth**	Superior frontal	−0.5	0.2	[−0.9, −0.07]	**0.04**	0.1	0.2	[−0.4, 0.5]	0.3
	Occipitotemporal	0.2	0.2	[−0.3, 0.7]	0.4	0.3	0.2	[−0.1, 0.8]	0.7
**Width**	Superior frontal	−0.1	0.2	[−0.5, 0.1]	0.3	0.1	0.1	[−0.2, 0.4]	0.5
	Occipitotemporal	0.1	0.1	[−0.1, −0.3]	0.3	−0.07	0.1	[−0.3, 0.1]	0.5
**Age interaction**									
**Depth**	Superior frontal	−0.02	0.03	[−0.07, 0.03]	0.5	−0.01	0.03	[−0.08, 0.05]	0.6
	Occipitotemporal	−0.07	0.03	[−0.1, −0.006]	0.08	−0.05	0.03	[−0.1, −0.002]	0.1
**Width**	Superior frontal	0.01	0.02	[−0.03, 0.05]	0.6	−0.03	0.02	[−0.06, 0.004]	0.2
	Occipitotemporal	0.03	0.01	[.003, 0.06]	0.058	0.008	0.01	[−0.02, 0.03]	0.5
**Age of first exposure**									
**Depth**	Superior frontal	0.01	0.04	[−0.08, 0.1]	0.8	−0.02	0.06	[−0.1, 0.09]	0.8
	Occipitotemporal	0.05	0.05	[−0.04, 0.1]	0.6	0.06	0.04	[−0.02, 0.1]	0.3
**Width**	Superior frontal	0.03	0.03	[−0.02, 0.09]	0.2	−0.04	0.03	[−0.09, 0.02]	0.3
	Occipitotemporal	−0.05	0.02	[−0.09, −0.01]	**0.03**	−0.02	0.02	[−0.05, 0.02]	0.3
**Total years in football**									
**Depth**	Superior frontal	−0.01	0.03	[−0.06, 0.05]	0.7	0.009	0.03	[−0.06, 0.07]	0.8
	Occipitotemporal	−0.04	0.03	[−0.09, 0.02]	0.4	−0.01	0.03	[−0.07, 0.04]	0.8
**Width**	Superior frontal	0.003	0.02	[−0.03, 0.04]	0.9	0.03	0.02	[−0.01, 0.08]	0.3
	Occipitotemporal	−0.03	0.01	[−0.06, −0.003]	**0.047**	−0.01	0.01	[−0.04, 0.01]	0.3
**CHII-Frequency (CHII)**									
**Depth**	Superior frontal	−0.000007	0.00003	[−0.00007, 000005]	0.8	−0.00004	0.00004	[−0.0001, 0.00003]	0.5
	Occipitotemporal	−0.00003	0.00003	[−0.00009, 0.00003]	0.5	0.00002	0.00003	[−0.00003, 0.00007]	0.5
**Width**	Superior frontal	0.00003	0.00002	[−0.00002, 0.00007]	0.5	−0.00002	0.00002	[.00007, 0.00002]	0.5
	Occipitotemporal	0.000001	0.00002	[−0.00003, 0.00004]	0.9	−0.0000002	0.00001	[−0.00003, 0.00003]	0.9
**CHII-Linear Acceleration (CHII-G)**									
**Depth**	Superior frontal	−0.000003	0.000001	[−0.000006, −0.0000007]	**0.03**	−0.000004	0.000002	[−0.000008, −0.0000002]	0.08
	Occipitotemporal	−0.000002	0.000002	[−0.000005, 0.000002]	0.4	−0.000001	0.000001	[−0.000004, 0.000002]	0.4
**Width**	Superior frontal	0.0000004	0.000001	[−0.000002, 0.000003]	0.7	0.0000002	0.000001	[−0.000002, 0.000002]	0.9
	Occipitotemporal	−0.0000004	0.0000009	[−0.000002, 0.000001]	0.7	−0.000001	0.0000006	[−0.000002, 0.0000003]	0.2
**CHII-Rotational Force (CHII-R)**									
**Depth**	Superior frontal	−0.00000003	0.00000002	[−0.00000007, 0.0000000004]	0.1	−0.00000005	0.00000002	[−0.00000009, −0.000000002]	0.09
	Occipitotemporal	−0.000000006	0.00000002	[−0.00000005, 0.00000003]	0.8	−0.000000001	0.00000002	[−0.00000004, 0.00000004]	0.9
**Width**	Superior frontal	0.00000000008	0.00000001	[−0.00000003, 0.00000003]	1	0.000000001	0.00000001	[−0.00000003, 0.00000003]	0.9
	Occipitotemporal	−0.000000006	0.00000001	[−0.00000003, 0.00000001]	1	−0.00000001	0.000000007	[−0.00000002, 0.000000006]	0.4
**TES**									
**Depth**	Superior frontal	−0.3	0.2	[−0.06, −0.003]	0.4	−0.2	0.3	[−0.8, 0.3]	0.8
	Occipitotemporal	−0.06	0.2	[−0.7, 0.2]	0.8	−0.06	0.2	[−0.5, 0.4]	0.8
**Width**	Superior frontal	−0.2	0.1	[−0.5, 0.1]	0.5	0.2	0.2	[−0.2, 0.5]	0.3
	Occipitotemporal	0.03	0.1	[−0.2, 0.3]	0.8	−0.2	0.1	[−0.4, 0.01]	0.1

Estimates, 95% CI, and *P* values for regions of interest for group-level differences and interactions. All *P* values are corrected for multiple comparisons. All significant values at *P* < 0.05 are bolded.

### Exposure to RHI and sulcal morphology

We identified a significant association between the age of first exposure to tackle football and left hemisphere occipitotemporal sulcal width (95% CI [−0.09, −0.01], *P* = 0.03), which showed wider sulcal width in those who participated in football at earlier ages; see Table 2. Additionally, we observed an association between the left hemisphere occipitotemporal sulcal width and total years in football (95% CI [−0.06, −0.003], *P* = 0.047), showing wider sulci the longer a football player participated in football. Moreover, we showed an association between left hemisphere superior frontal sulcal depth and CHII-G (95% CI [−0.000006, −0.0000007], *P* = 0.03), with shallower sulcal depth associated with increased estimates of linear acceleration throughout a football career; see Table 2. No other associations, including group-level associations with age, reached significance; all *P*’s > 0.058; see Table 2.

### Group-level differences in TES and provisional levels of certainty for CTE pathology

There were no differences in sulcal width (all *P*’s > 0.1) or sulcal depth (all *P*’s > 0.7) between our former American football players with or without a diagnosis of TES. Furthermore, there were no significant differences in sulcal width (all *P*’s > 0.5) or sulcal depth (all *P*’s > 0.07) across the levels of certainty for CTE pathology.

### Associations with neuropsychological evaluations

There was no significant association between the neuropsychological evaluations and sulcal width (all *P*’s > 0.5) or depth (all *P*’s > 0.6).

### Flortaucipir-PET

There was no significant association between flortaucipir SUVR values and sulcal width (all *P*’s > 0.1) or depth (all *P*’s > 0.4).

## Discussion

This study aimed to characterize *in vivo* sulcal morphometric changes in former American football players, focusing on the superior frontal and occipitotemporal regions associated with postmortem CTE neuropathology. We found that former players exhibited shallower sulcal depth in the left superior frontal region compared to controls. Additionally, earlier age of first exposure to tackle football and longer football participation were associated with wider occipitotemporal sulci, while greater cumulative head impact exposure correlated with shallower superior frontal sulci. Sulcal morphology was neither associated with age, TES, levels of certainty for CTE pathology, neuropsychological test performance, nor flortaucipir PET. We review the details of these findings below.

### Sulcal morphological changes in former American football players

Our results show evidence of shallower sulcal depth in the left hemisphere superior frontal region among former American football players compared to unexposed controls, suggesting a potential relationship between RHI and cerebral morphology.^61^ A hallmark feature of CTE is the accumulation of p-tau at the depths of the cortical sulci. The sulci appear to be particularly susceptible to RHI, a vulnerability supported by computational and physical models of brain biomechanics.^62,63^ These models demonstrate that the gyral architecture of the brain concentrates mechanical strain at the crests of gyri and, most notably, at the depths of sulci, regions also prone to the formation of cavitation vapor bubbles.^64,65^ This elevated strain may disrupt axonal integrity and contribute to the focal initiation of tau pathology. Over time, the accumulation of p-tau in these regions can promote neuroinflammation, neuronal loss and cortical atrophy, potentially resulting in measurable structural changes such as sulcal widening and shallowing. Thus, alterations in sulcal morphology may serve as an indirect *in vivo* marker of underlying p-tau deposition and associated neurodegenerative processes in individuals exposed to RHI.

This hypothesis is further supported by prior research demonstrating volume loss in CTE-associated brain regions in former American football players, particularly within the superior frontal region.^66^ These findings suggest that sulcal widening and shallowing may possibly reflect neurodegenerative processes linked to tau pathology, providing a potential structural biomarker for early detection and disease progression monitoring in individuals at risk for CTE. Yet, our findings highlight shallower sulcal depth in the left hemisphere superior frontal region but no differences in sulcal width. While most cases of CTE present with cortical p-tau accumulation, there are individuals exposed to RHI who develop CTE that show a low overall cortical burden of p-tau pathology, which is classified as cortical-sparing CTE.^23^ Therefore, it may be the case that if a sub-group of former American football players have CTE, some may have cortical-sparing CTE, making it even more difficult to detect robust findings in cortical areas. Importantly, since we do not have pathological confirmation of CTE in our sample, it is also possible that some of the athletes included in the study may not have CTE, which adds to the variability of our findings. Yet, our findings support the notion that shallower sulci are primarily a result of cortical atrophy, a phenomenon we have previously observed in this sample.^66^ This atrophy contributes to a reduction in sulcal depth, which we hypothesize is driven by the shrinkage of the adjacent gyri. As the gyri shrink, the sulci appear broader, thereby altering their apparent depth. This mechanism aligns with our previous reports, offering further insight into the relationship between cortical changes and now sulcal morphology. It is also important to note that the observed differences may be associated with the consequences of RHI and not necessarily indicative of CTE, as not everyone exposed to RHI will develop CTE. Therefore, these group-level differences observed in the sulci of our cohort of former American football players may be consequences of their exposure to RHI and unrelated to the neuropathology of CTE. These findings stress the need for further research to disentangle the specific effects of RHI from CTE neuropathology, as not all exposed individuals develop CTE.

### Associations between sulcal morphology and age

We did not observe an age-by-group interaction in sulcal width or depth in the superior frontal and occipitotemporal regions. This is noteworthy as alterations in sulcal morphology have been identified in several neurodegenerative diseases, particularly Alzheimer’s disease. Prior studies have consistently shown that sulcal widening and shallower depths are common in areas of the brain affected by Alzheimer’s pathology, such as the temporal lobe, and these changes are thought to reflect underlying cortical atrophy associated with disease progression.^19-22^ Specifically, wider sulci and reduced sulcal depth in Alzheimer’s disease may serve as indirect markers of neurodegeneration, with these structural alterations potentially linked to loss of grey matter volume in affected regions. Therefore, our findings, or lack thereof, in the superior frontal and occipitotemporal areas, may suggest that sulcal morphology changes in former American football players may follow a distinct pattern compared to what has been observed in Alzheimer’s disease. The absence of age-related interactions in sulcal morphology could indicate that the structural changes in football players are driven more by cumulative head trauma rather than age-related neurodegenerative processes, as seen in the Alzheimer’s disease literature. Future research is necessary to explore sulcal morphology across different neurodegenerative diseases which could provide valuable insights into the pathophysiological differences between possible CTE and Alzheimer’s disease, especially in cohorts with varying ages and brain injury histories.

### Associations between sulcal morphology and exposure factors

Our findings revealed an association between a younger age of first exposure to American football and sulcal widening in the left hemisphere occipitotemporal region. This change underscores the potential influence of early-life RHI on cerebral morphology later in life. The link between the age of first exposure to football and subsequent changes in brain structure and cognition has been previously documented.^67-72^ Taken together, structural neuroimaging markers could serve as valuable additions to existing imaging biomarkers for detecting neurodegenerative processes in athletes with a history of RHI exposure. Our study adds to this body of literature by identifying more cortical sulcal morphological changes associated with early exposure to RHI. However, it is important to acknowledge that some studies indicate a younger age of first exposure to American football does not correlate with post-career health outcomes.^73-79^ However, our findings are supported by the association between total years of football play and the left hemisphere occipitotemporal region, which indicates that more years of play leads to wider sulcal width in our football players. Yet, we acknowledge that further research is essential to elucidate the full extent of the relationship between early and longer exposure to American football and overall brain morphometry.

An association between CHII-linear acceleration over a career in American football and shallower superior frontal sulcal depth was also observed. This highlights the potential impact of biomechanics and linear forces of RHI on sulcal morphology. Biomechanical studies suggest that head impacts in American football strain the cortical grey matter/white matter boundary near the cerebral sulci, potentially triggering a harmful cascade of pathological changes in these areas.^63^ We hypothesize that this could result in the accumulation of p-tau in the depths of cortical sulci, as observed in CTE neuropathology, subsequently leading to alterations in sulcal morphology. Previous research from our team has also revealed that CHII metrics are associated with alterations in subcortical structures. For example, the relative length of a cavum septum pellucidum, a non-specific biomarker of CTE, tends to increase as estimates of CHII-rotational forces are experienced during play.^80^ These findings provide insights into the factors associated with non-specific biomarkers of CTE. However, additional research is needed to unravel the complex relationship between different RHI exposure factors and specific sulcal morphological changes. The borderline associations with total years in football and CHII-rotation forces suggest potential trends that warrant further exploration with larger sample sizes and larger distributions in exposure metrics. Therefore, prospective studies using accelerometer data should be considered.

This regional dissociation, where sulcal widening in the left occipitotemporal region is associated with age of first exposure and years of play, while sulcal depth in the left superior frontal sulcus is linked only to cumulative head impact burden, may reflect distinct patterns of vulnerability across cortical regions. One possibility is that the occipitotemporal cortex, which matures earlier during neurodevelopment, may be more sensitive to early-life and prolonged exposure to RHI. In contrast, the superior frontal region, involved in higher-order executive functions, may be more susceptible to cumulative biomechanical strain sustained over time. These divergent associations could also reflect region-specific trajectories of atrophy or compensatory mechanisms in response to different types of exposure. While speculative, such findings underscore the importance of examining multiple structural markers across brain regions to capture the heterogeneous effects of RHI. Future longitudinal studies are needed to clarify whether these regionally specific patterns represent true biological distinctions or are influenced by measurement variability or sample characteristics.

### Associations between sulcal morphology and TES diagnosis

While our study highlights structural changes, the absence of significant group-level differences in sulcal morphology between former American football players with or without TES underscores the need for comprehensive diagnostic criteria that include possible biomarkers. Reliance on patient history and clinical symptoms alone for TES diagnosis is a significant limitation. Efforts should continue to integrate biomarkers and supportive features into diagnostic criteria to enhance accuracy and reliability. This is particularly important because the clinical symptoms associated with TES diagnoses are not exclusive to possible CTE or RHI exposure; these symptoms also appear in the general population, especially among adults experiencing chronic pain, idiopathic mental health issues, or both.^81^ Additionally, cognitive and neurobehavioural symptoms are common among those who participate in contact and collision sports, but these symptoms are not always distinct between individuals with and without confirmed CTE.^24,82^ Our investigation indicates that further studies examining the relationship between TES and other potential *in vivo* biomarkers of CTE, as well as changes associated with RHI exposure, are warranted. This finding was further corroborated by the absence of any significant differences between our football players across varying levels of certainty for CTE pathology. Despite the use of criteria specifically designed to reflect increased clinical severity potentially linked to underlying p-tau pathology, this approach did not effectively differentiate between our groups. This suggests that the criteria intended to capture subtle variations in pathology did not yield the expected distinctions, highlighting the complexity of using clinical indicators to distinguish between varying levels of neurodegenerative progression.

### Associations between sulcal morphology and neuropsychological evaluation

Given the absence of group-level differences in TES diagnosis or the levels of certainty for CTE pathology, we performed a post-hoc investigation of how sulcal morphological changes may be associated with neuropsychological evaluations. It is also important to highlight that the superior frontal and occipitotemporal regions, selected a priori, are critically linked to cognitive domains such as executive function, working memory, and attention—key components of TES diagnosis and common cognitive complaints among former contact–collision sports athletes. By focusing on these regions, we sought to capture more relevant, quantifiable cognitive impairments that may better reflect the sulcal morphometric differences we observed, providing a more comprehensive understanding of the underlying structural changes. However, we did not find any significant associations between sulcal morphology and performance on neuropsychological assessments. This suggests that variations in these structural features may not directly influence cognitive function, at least within the scope of our study. The relationship between sulcal morphology and cognitive outcomes remains complex and challenging to pinpoint, as sulcal changes may reflect broader neuroanatomical processes that interact with multiple structural and functional brain networks. Additionally, cognitive function is influenced by a multitude of factors, including white matter integrity, cortical thickness and connectivity patterns, which may play a more direct role than sulcal morphology alone.^20,83-88^ Further research incorporating multimodal imaging and longitudinal analyses is needed to understand better how sulcal characteristics contribute to cognitive aging and disease progression in individuals at higher risk of developing CTE.

### Associations between sulcal morphology and flortaucipir-PET

Lastly, our study also explored the association between sulcal morphology and flortaucipir accumulation detected via PET, and no significant associations were observed between sulcal morphology and flortaucipir SUVR values. Previous findings using the same sample of former American football players found significantly elevated flortaucipir uptake in former American football players bilaterally in the superior frontal, bilateral medial temporal and left parietal regions.^11^ This study also reported no differences in flortaucipir SUVR values in football players with or without a TES diagnosis.^11^ Sulcal morphology may represent a late-stage indicator of cortical damage following the accumulation of p-tau. Given this hypothesized difference in timing, a significant association may not be observed in this cross-sectional analysis. Future longitudinal studies should explore the relationship between cortical flortaucipir uptake and the development of alterations in sulcal morphology. Additionally, in this study, it may be the case that sulcal morphology detected via structural MRI may not be sufficiently sensitive to show a significant correlation with PET data. This lack of association suggests that structural changes in the sulci assessed may not directly mirror tau deposition, but rather the consequences of tau accumulation like aging or atrophy, potentially highlighting the complexity of the relationship between structural alterations and molecular biomarkers.

### Limitations

Some limitations need to be considered. First, the cross-sectional design restricts causal inferences, emphasizing the need for longitudinal studies to clarify the temporal dynamics of sulcal changes. Second, the absence of postmortem data prevents us from directly linking our findings to the underlying pathology. Third, the inclusion of only male former American football players who participated between 1952 and 2007 affects the generalizability of the findings. This time span also limits our scope, given the evolution of football in terms of play intensity and health protocols. Future studies should aim to include larger cohorts that include participants from other sports, gender identities, and participation timeframes. Fourth, our unexposed control participants were asymptomatic at the time of screening, which could influence group-level comparisons as it limits our comparison to those who do not show cognitive deficits either as a sign of natural aging or other neurodegenerative diseases, like Alzheimer’s disease. Fifth, although a substantial number of our football players and unexposed controls identify as Black, mirroring the demographics of former NFL players from 1967 to 1996, future studies should attempt to recruit a more diverse sample of participants to reflect the current demographical breakdown of the NFL. Sixth, our methodology is limited by the regions assessed using CalcSulc, as this measurement tool restricts the examination of other brain regions implicated in CTE neuropathology. Furthermore, our analysis of the association between sulcal morphology and flortaucipir-PET employs two distinct segmentation atlases (Destrieux for CalcSulc and Desikan–Killiany for flortaucipir SUVR calculations). This methodological complexity might explain the lack of observed associations, as the two regions do not fully overlap. Nonetheless, we used the closest matching brain regions from each atlas for the best possible correspondence. Lastly, and seventh, the lack of helmet accelerometer data from professional football players for deriving CHII scores introduces not only variability, but it is important to note that the scores were estimated from self-reported data on seasons played, player positions and accelerometer data from youth, high school and collegiate athletes. Thus, they are estimates of head impact exposure and do not capture the individual variability in head impact exposure. Thus, future studies are needed that incorporate long-term helmet accelerometer data.

## Conclusion

In summary, this study reports shallower sulcal depths in former American football players in the superior frontal region, a brain region commonly affected in the initial stages of CTE, compared to unexposed asymptomatic controls. We also observed interactions between sulcal morphometric measures (depth and width) and RHI exposure metrics (age of first exposure to football, total years in football and career-long estimates of linear acceleration). However, contrary to our hypotheses, we did not find significant group-level differences between football players with and without a TES diagnosis or varying levels of certainty for CTE pathology. Additionally, we found no significant associations between sulcal morphology measures and age, neuropsychological assessments, and flortaucipir-PET uptake. Overall, our findings could indicate that exposure to RHI impacts the morphometric properties of the sulci, with earlier and greater exposure associated with more pronounced brain alterations later in life.

## Supplementary Material

fcaf345_Supplementary_Data

## Data Availability

Data from the DIAGNOSE CTE Research Project will be available to qualified investigators through the Federal Interagency Traumatic Brain Injury Research (FITBIR) Informatics System, through the National Institutes of Health (NIH) Center for Information Technology: https://fitbir.nih.gov/content/access-data. DIAGNOSE CTE Research Project data, including those reported in this study, will also be available to qualified investigators through a project-specific data-sharing portal. The data and research tools utilized in this manuscript were obtained and analysed from controlled access datasets provided by the DOD- and NIH-supported Informatics Systems. Established by the Department of Defense and the National Institutes of Health, this Informatics System serves as a national resource to advance research in TBI and trauma. Dataset identifier: DOI. The views expressed in this manuscript are those of the authors and do not necessarily represent the opinions or perspectives of the DOD, NIH, or the original data submitters to the Informatics System. For specific questions regarding the data presented in this manuscript, interested investigators should contact Dr. Hector Arciniega at Hector.Arciniega@nyulangone.org.

## Appendix

### DIAGNOSE CTE Research Project Team

Eric Reiman, M.D., Yi Su, Ph.D., Kewei Chen, Ph.D., Hillary Protas, Ph.D., Connie Boker, M.B.A., Michael L. Alosco, Ph.D., Rhoda Au, Ph.D., Robert C. Cantu, Ph.D., Lindsay Farrer, Ph.D., Robert Helm, M.D., Douglas I. Katz, M.D., Neil Kowall, M.D., Jesse Mez, M.D., Gustavo Mercier, M.D., Ph.D., James Otis, M.D., Robert A. Stern, Ph.D., Jason Weller, M.D., Irene Simkin, M.S., Alondra Andino, B.A., Shannon Conneely, B.A., Courtney Diamond, M.B.A., Tessa Fagle, B.A., Olivia Haller, B.A., Tennyson Hunt, M.B.A., Nicole Gullotti, M.B.A., Megan Mariani, B.S., B.A., Brian Mayville, B.S., Kathleen McLaughlin, B.A., Mary Nanna, B.A., Taylor Platt, M.P.H., Surya Pulukuri, B.A., Fiona Rice, M.P.H., Madison Sestak, B.S., Michael McClean, Sc.D., Yorghos Tripodis, Ph.D., Douglas Annis, M.S., Christine Chaisson, M.P.H., Diane B. Dixon, Carolyn Finney, B.A., Kerrin Gallagher, M.P.H., Kaitlin Hartlage, M.P.H., Jun Lu, M.S., Brett Martin, M.S., Emmanuel Ojo, M.P.H., Joseph N. Palmisano, M.A., M.P.H., Brittany Pine, B.A., B.S., Janani Ramachandran, M.S., Sylvain Bouix, Ph.D., Jennifer Fitzsimmons, M.D., Alexander P. Lin, Ph.D., Inga K. Koerte, M.D., Ph.D., Ofer Pasternak, Ph.D., Martha E. Shenton, Ph.D., Hector Arcinieago, Ph.D., Tashrif Billah, M.S., Elena Bonke, M.S., Katherine Breedlove, Ph.D., Eduardo Coello, Ph.D., Michael J. Coleman, M.A., Leonhard Jung, Huijun Liao, B.S., Maria Loy, M.B.A., M.P.H., Elizabeth Rizzoni, B.A., Vivian Schultz, M.D., Annelise Silva, B.S., Brynn Vessey, B.S., Tim L.T. Wiegand, Sarah Banks, Ph.D., Charles Bernick, M.D., Jason Miller, Ph.D., Aaron Ritter, M.D., Marwan Sabbagh, M.D., Raelynn de la Cruz, Jan Durant, Morgan Golceker, Nicolette Harmon, Kaeson Kaylegian, Rachelle Long, Christin Nance, Priscilla Sandoval, Robert W. Turner, Ph.D., Kenneth L. Marek, M.D., Andrew Serrano, M.B.A., Charles H. Adler, M.D., Ph.D., David W. Dodick, M.D., Yonas Geda, M.D., MSc, Jennifer V. Wethe, Ph.D., Bryce Falk, R.N., Amy Duffy, Marci Howard, Michelle Montague, Thomas Osgood, Debra Babcock, M.D., Ph.D., Patrick Bellgowan, Ph.D., Laura Balcer, M.D., M.S.C.E., William Barr, PhD., Judith Goldberg, Sc.D., Thomas Wisniewski, M.D., Ivan Kirov, Ph.D., Yvonne Lui, M.D., Charles Marmar, M.D., Lisena Hasanaj, Liliana Serrano, Alhassan Al-Kharafi, Allan George, Sammie Martin, Edward Riley, William Runge, Jeffrey L. Cummings, M.D., ScD, Elaine R. Peskind, M.D., Elizabeth Colasurdo, Daniel S. Marcus, Ph.D., Jenny Gurney, M.S., Richard Greenwald, Ph.D. and Keith A. Johnson, M.D.
