# Supplementary material for: Sulcal morphology in former American football players
Source: Brain Commun. 2025 Sep 11;7(5):fcaf345. doi: 10.1093/braincomms/fcaf345 (PMC12492488; doi:10.1093/braincomms/fcaf345)
Supplement: fcaf345_Supplementary_Data [file fcaf345_supplementary_data.docx]

| **Inclusion** | **Exclusion** |
| --- | --- |
| **Overall Criteria** | |
| - Male, age 45-74 - No contraindications to procedures performed in the DIAGNOSE CTE Research Project - English as primary language - Willingness and ability to consent to study participation of the participants and a study partner | - History of clinical Stroke or significant neurological condition, severe enough to compromise neuropsychological testing - Other clinically significant organ disease (e.g. liver, kidney…) |
| **Former professional American football players** | |
| - ≥12 years of organized football (≥3 in college; ≥4 in the NFL) | - Not quarterback, kicker, punter or special teams only |
| **Former collegiate American football players** | |
| - ≥6 years of organized football (≥3 in college) | - Not quarterback, kicker, punter or special teams only |
| **Unexposed Participants** | |
| - No history of participation in organized contact/collision sport or military service and training - Asymptomatic based on telephone screening regarding neuropsychological and psychiatric conditions - At least two years of secondary education | - History of TBI, Concussion - History of formal diagnosis of psychiatric or neurological illness |
| **Advertisement**  All Participants: Newspaper advertisement, billboard advertisement and social media posts.  Football targeted advertisement via NFL alumni groups and videos of Super Bowl champion Ben Utrecht. | |

| **DIAGNOSE CTE Research Project Sample**  (Cross-Sectional) | | |
| --- | --- | --- |
| N = 180 Former American football players | | N = 60 Unexposed Participants |
| N = 120 Former professional  football players | N = 60 Former collegiate   football players |  |

| **Exclusion due to data quality control** | |
| --- | --- |
| N = 11 Former American football players   - N = 11 (Missing or poor quality T1w MRI) | N= 6 Unexposed Participants   - N = 2 (Missing T1w MRI) - N = 2 (Undisclosed RHI exposure) - N = 2 (Undisclosed excluding  illness) |

| **Study Sample**  (Cross-Sectional) | | |
| --- | --- | --- |
| N = 169 Former American football players | | N = 54 Unexposed Participants |
| N = 114 Former professional  football players | N = 55 Former collegiate   football players |  |

**Supplementary Figure 1 Description:**
Inclusion and exclusion criteria of the DIAGNOSE CTE research project and the data included in this study. Abbreviations: NFL, National Football League; TBI, traumatic brain injury; MRI, magnetic resonance imaging.

|  | Inclusion Criteria | Exclusion Criteria |
| --- | --- | --- |
| All Participants [N=240] | - Male - Age Range 45-74 - English is the Primary Language - No MRI, Lumbar Puncture, or PET risk factors procedures - Agreement to all procedures performed - Willingly agrees and has available study partner who knows the participant well, speaks or meets with participant at least weekly for 6 months, is at least 18 years of age, and primary language is English (study partner agrees to answer online questionnaires) | - History of clinical stroke or other neurological conditions - Severe vision or hearing impairment that can potentially interfere with neuropsychological testing - Trouble making decisions affecting ability to provide informed consent - Presence of clinically significant infectious, endocrine, pulmonary, kidney, liver function, cancer diagnosis, or metabolic disease - Body weight exceeding 400 pounds |
| Unexposed Asymptomatic Control Group [N=60] | - No history of any participation in organized contact/collision sports, military combat service, or training - Asymptomatic, determined by screening questions (Evaluating current mood, behavior, cognitive symptoms, and functional dependence) - At least 2 years of post-secondary education at a 4-year accredited college/university (Or has an Associate’s degree) | - History of any Traumatic Brain Injury or Concussion - History of any formal diagnosis or treatment of psychiatric illness or cognitive impairment - A body mass index of less than 24 |
| Former College Football player group (COL) [N=60] | - Played for more than or equal to 6 years of organized American football including more than or equal to 3 years of college varsity level play - Played at least one of the following positions in college: offensive lineman, defensive lineman, linebacker, offensive back or receiver, or defensive back - No organized American football or contact/collision sports following college | - Primary athletic position was quarterback, or kicker |
| Former professional football player group (PRO) [N=120] | - Played for more than or equal 12 years of organized football this includes (more than or equal to 3 years in college and more than 3 years in the NFL) - Played at least one of the following positions: offensive lineman, defensive lineman, linebacker, offensive back or receiver, or defensive back | - Primary athletic position was quarterback, or kicker |

**Supplementary Table 1. Inclusion and exclusion criteria.**

|  |  | Left | |  | Right | |
| --- | --- | --- | --- | --- | --- | --- |
| **Region of Interest** | Estimate | SD | 95% CI | Estimate | SD | 95% CI |
| Superior Frontal | -.4 | .2 | [-.9, .006] | .2 | .3 | [-.4, .7] |
| Inferior Frontal | -.3 | .3 | [-.9, .2] | .3 | .3 | [-.4, .9] |
| Marginal Part of Cingulate | .04 | .2 | [-.3, .5] | .1 | .2 | [-.3, .6] |
| Central | -.3 | .3 | [-.8, .3] | -.2 | .3 | [-.8 .4] |
| Post-Central | -.3 | .4 | [-1.1, .3] | -.2 | .4 | [-1.0, .6] |
| Parieto-Occipital | -.3 | .4 | [-1.1, .4] | -.2 | .1 | [-1.0, .6] |
| Middle Occipital and Lunate | -.2 | .3 | [-.8, .4] | -.3 | .2 | [-.2, .8] |
| Occipitotemporal | .04 | .2 | [-.3, .7] | .3 | .2 | [-.2, .8] |

**Supplementary Table 2. Group differences for sulcal depth.** Estimates and 95% CI for all CalcSulc regions for the group differences between former American football players and unexposed asymptomatic controls. All p’s > .05.

|  |  | Left | |  | Right | |
| --- | --- | --- | --- | --- | --- | --- |
| **Region of Interest** | Estimate | SD | 95% CI | Estimate | SD | 95% CI |
| Superior Frontal | -.2 | .2 | [-.5, .1] | .2 | .1 | [-.1, .4] |
| Inferior Frontal | -.1 | .16 | [-.2, .4] | .2 | .2 | [-.1, .4] |
| Marginal Part of Cingulate | -.1 | .08 | [-.3, .07] | -.2 | .1 | [-.4, -.003] |
| Central | -.02 | .1 | [-.2, .3] | -.1 | .1 | [-.3 .1] |
| Post-Central | -.2 | .1 | [-.5, .1] | -.1 | .1 | [-.3, .1] |
| Parieto-Occipital | -.2 | .1 | [-.5, .01] | -.1 | .1 | [-.4, .1] |
| Middle Occipital and Lunate | -.01 | .1 | [-.2, .2] | -.2 | .1 | [-.4, .05] |
| Occipitotemporal | -.05 | .1 | [-.2, .3] | -.1 | .1 | [-.3, .1] |

**Supplementary Table 3. Group differences for sulcal width.** Estimates and 95% CI for all CalcSulc regions for the group differences between former American football players and unexposed asymptomatic controls. All p’s > .05.
